# A Feasibility Analysis of Land-Based SINS/GNSS Gravimetry for Groundwater Resource Detection in Taiwan

**DOI:** 10.3390/s151025039

**Published:** 2015-09-29

**Authors:** Kai-Wei Chiang, Cheng-An Lin, Chung-Yen Kuo

**Affiliations:** Department of Geomatics, National Cheng Kung University, No. 1, University Road, Tainan 701, Taiwan; E-Mails: kwchiang@mail.ncku.edu.tw (K.-W.C.); p68011068@mail.ncku.edu.tw (C.-A.L.)

**Keywords:** land-based SINS/GNSS gravimetry, gravity disturbance, groundwater resource detection

## Abstract

The integration of the Strapdown Inertial Navigation System and Global Navigation Satellite System (SINS/GNSS) has been implemented for land-based gravimetry and has been proven to perform well in estimating gravity. Based on the mGal-level gravimetry results, this research aims to construct and develop a land-based SINS/GNSS gravimetry device containing a navigation-grade Inertial Measurement Unit. This research also presents a feasibility analysis for groundwater resource detection. A preliminary comparison of the kinematic velocities and accelerations using multi-combination of GNSS data including Global Positioning System, Global Navigation Satellite System, and BeiDou Navigation Satellite System, indicates that three-system observations performed better than two-system data in the computation. A comparison of gravity derived from SINS/GNSS and measured using a relative gravimeter also shows that both agree reasonably well with a mean difference of 2.30 mGal. The mean difference between repeat measurements of gravity disturbance using SINS/GNSS is 2.46 mGal with a standard deviation of 1.32 mGal. The gravity variation because of the groundwater at Pingtung Plain, Taiwan could reach 2.72 mGal. Hence, the developed land-based SINS/GNSS gravimetry can sufficiently and effectively detect groundwater resources.

## 1. Introduction

According to the Taiwan Central Weather Bureau [1] and Water Resources Agency [2], the annual precipitation with an average of 62,000 mm per year in Taiwan is considered to be abundant. However, the supply of water to humans, agriculture, and industry is deficient because the water flows to the oceans directly and quickly because the topography is extremely steep. An alternative water supply source is to pump groundwater during surface water shortages. Searching for potential groundwater areas can be difficult, particularly when new or more available water resources may be stored deep underground. Gravity changes are caused directly by mass variations, and hence, fill thickness could produce subtle anomalies in the Earth’s gravitational field, which can be used to estimate fill thickness and bedrock topography if properly detected. Along these lines, gravity measurements have the potential for use in the detection or monitoring groundwater resources [3].

Conventional methods for measuring gravity changes include the use of terrestrial gravimeters and astronomical techniques. Although both methods can provide accurate gravity information with high resolution, the required instruments are extremely expensive and the field work is both labor- and time-consuming. Airborne and marine gravimeters have been developed to improve the efficiency of field work, however, the instruments are still expensive. Based on the circumstances in Taiwan, not only is the topography complicated and steep but also flight altitude is restricted by Taiwanese laws, so the flight altitude cannot be low. The gravity accuracy observed by airborne gravimeters decreases because of the downward continuation of gravity from the flight altitude to ground level. At the same time, the performance of marine gravimeters was also relatively poor due to ship vibrations.

With the advances in space technology, the global gravity field can be efficiently determined using satellite missions, such as Gravity Recovery and Climate Experiment and Gravity Field and Steady-state Ocean Circulation Explorer (GOCE). However, satellite missions cannot observe short wavelength gravity fields. For example, the gravity field derived from the measurements of the GOCE mission launched in 2009 has an accuracy of 1 mGal ($10^{-5}\;\text{m}/{\text{s}}^{2}$) with a spatial resolution of 100 km [4].

Strapdown Inertial Navigation System (SINS) and Global Navigation Satellite System (GNSS) have been integrated for navigation and kinematic positioning over the past few decades. An Inertial Navigation System (INS) contains an onboard computer and an Inertial Measurement Unit (IMU), consisting of three-axis accelerometers and three-axis gyroscopes used to determine the motion of the vehicle and provide full navigation states. Benefiting from the improvements in IMU hardware and GNSS kinematic positioning technology, the moving base SINS/GNSS gravimetry has been developed and proven successfully to perform well in estimating gravity. Schwarz [5] presented the equations for estimating gravity vectors based on GNSS-derived kinematic acceleration and IMU-measured dynamic acceleration. Airborne SINS/GNSS gravimetry can locally augment low resolutions (>150 km) to medium information (5 to 150 km), and land-based gravimetry can be increase to high resolution information (<5 km) [4]. The moving base SINS/GNSS gravimetry is an effective and mature method for surveying local gravity within a relatively short period, and can now be used in geophysical and geodetic applications.

However, because of the attenuation of the gravity field with altitudes, detecting short wavelength gravity signals has become a challenge. Considering that the power of the gravity field intensifies as the altitude decreases, the signal-to-noise ratio (SNR) should increase in the land-based system. The lower velocity of the land-based vehicle can also improve the resolution and accuracy of gravity disturbance [6], which is defined as the difference between the actual gravity and the normal gravity. In general, the high resolution gravity disturbance based on the smoothing of 180 s can be obtained with an accuracy of 3 mGal in a vertical component [6].

Land-based SINS/GNSS gravimetry, which has better mobility and flexibility, can serve as a good alternative. Hence, to increase the SNR and avoid the errors of a downward continuation process, in the current study a prototype of land-based gravimetry based on SINS/GNSS is developed for groundwater detection.

## 2. Problem Statements

Moving base gravimetry is constructed by integrating GNSS and INS. GNSS can provide precise positioning and kinematic acceleration, whereas INS can measure the specific forces encountered by the vehicle. In general, INS has two kinds of mechanization for INS namely, gimbaled INS and SINS [7].

Gimbaled INS is a physical realization of the navigation-frame (*n*-frame) that uses a gyro-stabilized platform with three orthogonal accelerometers. Thus, the accelerometers and gyroscopes do not encounter kinematic measurement errors directly. The configurations may lead to good gravity estimates [8], but the shortcomings are that the platform has a relatively large size and is expensive. Compared with gimbaled INS, SINS can provide an analytical image of the *n*-frame, and the accelerometers and gyroscopes are installed in the unit. The advantages of SINS are its smaller size, lower cost, and more operational flexibility [9]. Therefore, the moving base SINS/GNSS gravimetry is developed; the gravity disturbance from this system can achieve an accuracy of 3 to 5 mGal [10].

**Figure 1:**
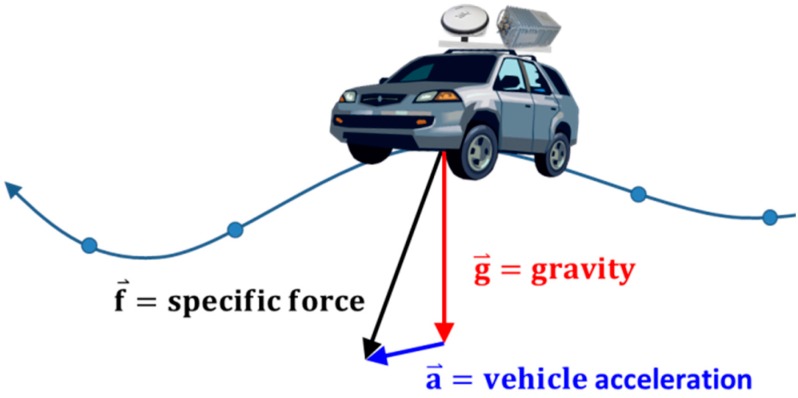
Concept of land-based SINS/GNSS gravimetry.

In the SINS/GNSS gravimetry technique, as shown in Figure 1, the IMU accelerometers measure the specific force along the trajectory, whereas the GNSS provides the kinematic acceleration of the vehicle. In principle, the measured specific force contains gravity and the effects of the motion. According to Newton’s second law of motion, in a non-rotating coordinate frame, gravity can be derived from the difference between the specific force and kinematic acceleration.

In practice, the extremely low SNR is the primary problem that needs to be addressed in determining the gravity signal. Representatively, the magnitude of gravity disturbance does not exceed 100 mGal in the vertical component over the distance of about 100 km [11]. However, non-gravitational accelerations measured by SINS/GNSS could be over 100 to 1000 times larger than the gravity disturbance [12,13]. A direct and easy method of making the gravity disturbance signal more noticeable from measurement errors is to decrease the altitude of the vehicle [6].

The first purpose of this research is to implement a land-based SINS/GNSS gravimetry method that can provide optimal estimations of gravity disturbance because of its low altitude compared with airborne SINS/GNSS gravimetry. The main idea is that gravity disturbance can be obtained directly by differencing the GNSS-derived kinematic acceleration and IMU-measured dynamic acceleration. Compared with the traditional method, the computations in this research can be carried out in both the inertial-frame (*i*-frame) and the *n*-frame, thereby avoiding a significant amount of computations related to the equations of the error dynamics applied in the traditional method.

In terrestrial tests, both IMU and GNSS measurements have much larger noises because of the rough terrain and vehicle vibrations. Thus, a low-pass filter is required to smooth the data. High resolution gravity disturbance with an accuracy of 3 mGal in a vertical component based on the smoothing of 180 s can be obtained [14]. Accuracy and resolution do not only rely on the algorithms of the data processing, but rather rely mostly on the performance characteristics of the IMU, velocity of the vehicle, altitude, and quality of the positioning solutions [15]. High-end navigation grade IMU, moderate velocity, low altitude, and cm-level positioning solutions are required to obtain accurate results of SINS/GNSS gravimetry. However, system configurations may not be always ideal during tests. Hence, detecting gravity signals with sufficient accuracies may be meaningful and worthwhile for novel studies.

The accurate determinations of a vehicle’s velocity and kinematic acceleration using GNSS are often acquired in a moving base SINS/GNSS gravimetry. In turn, the differentiation of GNSS signals is a crucial procedure for obtaining estimates and is important in post-processing where the acceleration is estimated conventionally by differentiating GNSS positions [16]. High precision results can now be achieved because of the evolution of GNSS. Hence, the second purpose of this study is to evaluate kinematic velocity and acceleration using Multi-GNSS, including Global Positioning System (GPS), Global Navigation Satellite System (GLONASS), and BeiDou Navigation Satellite System (BDS).

## 3. Gravity Determination Derived from SINS/GNSS Gravimetry

IMU measures the dynamic acceleration directly applied on lifting and dragging, and thus, measured gravity is retrievable from the kinematic acceleration computed from the GNSS position [15]. SINS/GNSS gravimetry can be estimated after obtaining the determination of the measured gravity. The principle equation in the *n*-frame is described in the following [17]:

(1) $${\text{δg}}^{\text{n}}={{\text{a}}_{u}}^{\text{n}}-{\ddot{{\text{x}}_{u}}}^{\text{n}}-{\text{γ}}^{n}+\left(\frac{{\text{V}}_{e}}{{\text{R}}_{N}+\text{h}}+2{\text{ω}}_{E}\text{cos}\varphi \right){\text{V}}_{e}+\frac{{{\text{V}}_{n}}^{2}}{{\text{R}}_{M}+\text{h}}$$

where γ is the normal gravity, ${\text{V}}_{e}$ and ${\text{V}}_{n}$ are the east and north components of the vehicle’s velocity, respectively, ${\text{ω}}_{E}$ is the rotation rate of Earth, h and φ are the ellipsoidal height and geodetic latitude, and ${\text{R}}_{N}$ and ${\text{R}}_{M}$ are prime and meridian curvature radii, respectively. The sum of the fourth and fifth terms at the right side is usually called the Eötvös correction, which results from the rotation of the reference coordinate frame.

In this research, all calculations are performed in the *i*-frame. Therefore, it is conceptually simpler, more straightforward and computationally less compared to the traditional approach (Equation (1)). The basic equation is based on Newton’s second law of motion expressed in a non-rotating and free falling coordinate frame (*i*-frame) [7], which reads as follows:

(2) $${\text{g}}^{i}={{\text{a}}_{u}}^{\text{i}}-{\ddot{{\text{x}}_{u}}}^{\text{i}}$$

where g is the measured gravity, $\ddot{{\text{x}}_{\text{u}}}$ is the vertical kinematic acceleration from the upward component, and ${\text{a}}_{u}$ is the dynamic acceleration.

The principal algorithms of the proposed SINS/GNSS gravimetry are divided into three parts, including the lever-arm correction, GNSS-derived kinematic acceleration, and IMU-measured dynamic acceleration.

### 3.1. Lever-Arm Correction

Because the phase center of the GNSS antenna does not coincide with the fiducial center of the IMU, the kinematic accelerations computed from the GNSS positions cannot be compared directly with the IMU-measured dynamic accelerations. The displacement vector between the GNSS antenna and the IMU is called the lever-arm, and the GNSS positions should be corrected to ensure that the lever-arm effect obtains reasonable gravimetry results [7]. Supposing that the GNSS position of the antenna is obtained in *i*-frame, then the position of the IMU in *i*-frame is given by:

(3) $${\text{X}}_{\text{IMU}}^{\text{i}}={\text{X}}_{\text{GNSS antenna}}^{\text{i}}-{\text{C}}_{b}^{\text{i}}{\text{L}}^{b}$$

where ${\text{L}}^{b}$ is the lever-arm displacement vector in *b*-frame and ${\text{C}}_{b}^{\text{i}}$ is the rotation matrix transforming ${\text{L}}^{b}$ in *b*-frame to that in *i*-frame. The rotation matrix is computed in terms of geodetic latitude φ and longitude λ:

(4) $${\text{C}}_{b}^{\text{i}}={\text{C}}_{n}^{\text{i}}\times {\text{C}}_{b}^{\text{n}}$$

(5) $${\text{C}}_{n}^{\text{i}}={\text{C}}_{b}^{\text{i}}\times {\left({\text{C}}_{b}^{\text{n}}\right)}^{-1}=\left[\begin{array}{ccc} -\text{sin}\left(\text{φ}\right)\text{cos}\left(\text{θ}+\text{λ}\right) & -\text{sin}\left(\text{θ}+\text{λ}\right) & -\text{cosφcos}\left(\text{θ}+\text{λ}\right)\\ -\text{sin}\left(\text{φ}\right)\text{sin}\left(\text{θ}+\text{λ}\right) & \text{cos}\left(\text{θ}+\text{λ}\right) & -\text{cosφsin}\left(\text{θ}+\text{λ}\right)\\ \text{cosφ} & 0 & -\text{sinφ} \end{array}\right]$$

where $\text{θ}={\text{ω}}_{E}\left(\text{t}-{\text{t}}_{0}\right)$, and ${\text{t}}_{0}$ is an initial epoch when the inertial and Earth-centered and Earth-fixed (ECEF) coordinate frames are assumed to be parallel. The initial epoch is usually chosen as the first epoch when both GNSS and INS data are available. The INS epoch closest to the GNSS epoch must be used because the sampling rates differ.

### 3.2. Kinematic Acceleration

Precise GNSS positioning solutions can be processed using a standard differential technique based on the phase observation from GNSS receivers on the moving vehicle and at a number of base stations. Conventionally, the GNSS positions ${\text{X}}^{e}$ are given in the ECEF coordinate frame (*e*-frame). However, to reduce the computational complexity, the transformation of the GNSS positions from *e*-frame to *i*-frame should be performed before the differentiation. The equation can be expressed as:

(6) $${\text{X}}^{i}={\text{C}}_{e}^{\text{i}}{\text{X}}^{e}=\left[\begin{array}{ccc} \text{cos}\left(-\omega_{\text{E}}\text{t}\right) & \text{sin}\left(-\omega_{\text{E}}\text{t}\right) & 0\\ -\text{sin}\left(-\omega_{\text{E}}\text{t}\right) & \text{cos}\left(-\omega_{\text{E}}\text{t}\right) & 0\\ 0 & 0 & 1 \end{array}\right]{\text{X}}^{e}$$

where ${\text{C}}_{e}^{\text{i}}$ is a matrix that rotates the coordinate frame of ${\text{X}}^{e}$ along the third axis by the angle $\omega_{\text{E}}\text{t}$ and t is time with respect to the reference epoch.

The kinematic acceleration of the vehicle can be determined by the numerical differentiation of the precise GNSS positions corrected by the lever-arm displacement:

(7) $${\ddot{\text{X}}}_{\text{IMU}}^{\text{i}}=\frac{{\text{d}}^{2}}{{\text{dt}}^{2}}\left({\text{X}}_{\text{GNSS antenna}}^{\text{i}}-{\text{C}}_{b}^{\text{i}}{\text{L}}^{b}\right)$$

The smoothing process, which reduces the noise to the level of a few mGal, should be applied to obtain accurate kinematic accelerations.

### 3.3. Dynamic Acceleration

The dynamic accelerations sensed by the IMU are referenced to the platform frame because the SINS is mounted to the strapdown mechanization platform. Hence, to obtain dynamic accelerations in *i*-frame, the transformation should be applied according to:

(8) $${\text{a}}^{i}={\text{C}}_{b}^{\text{i}}{\text{a}}^{b}$$

The transformation matrix ${\text{C}}_{b}^{\text{i}}$ is calculated from the gyroscope output using the optimal estimates from Rauch-Tung-Striebel (RTS) smoother [18] with the aid of GNSS. The dynamic acceleration ${\text{a}}^{b}$ is obtained after correcting systematic errors (bias and scale factor), which are also estimated using the RTS smoother. Therefore, the measured gravity determined by the proposed SINS/GNSS gravimetry is based on the following:

(9) $${\text{g}}^{n}={\text{C}}_{i}^{\text{n}}\left({{\text{a}}_{u}}^{\text{i}}-{\ddot{{\text{x}}_{\text{u}}}}^{\text{i}}\right)$$

where the transformation matrix ${\text{C}}_{i}^{\text{n}}$ can be obtained by transposing ${\text{C}}_{n}^{\text{i}}$. The starting points in the epoch for the smoothing of both GNSS and INS data must be the same and thus, the difference between kinematic and dynamic accelerations should be smoothed.

## 4. Implementation of Proposed Land-Based SINS/GNSS Gravimetry

**Figure 2:**
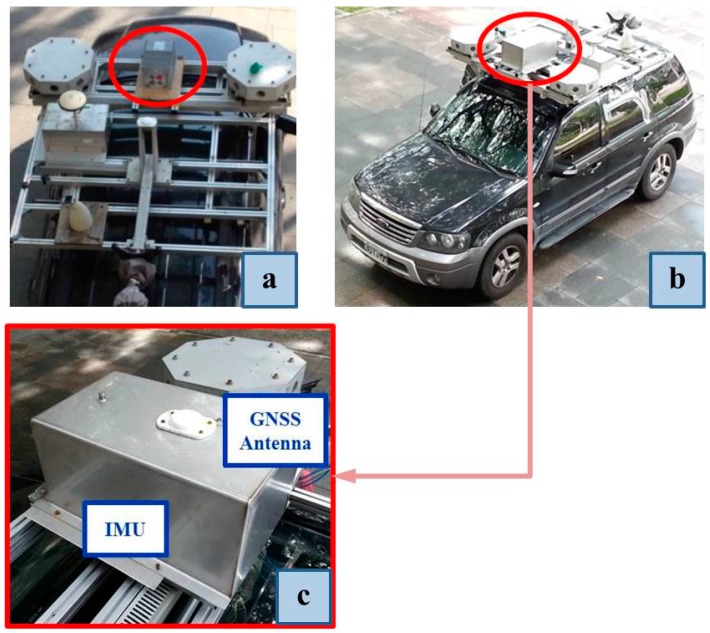
(**a**) Setup of sensors applied for the land-based system. (**b**) A steel box used for protecting IMU and the GNSS antenna was installed on top of its. (**c**) A zoom-in view of (b).

Figure 2a shows our land-based system which was used for terrestrial experiments and the IMU was just mounted on top of the vehicle without the steel box. In Figure 2b, the IMU was enclosed in a steel box for protection and the GNSS antenna was installed on top of the steel box to prevent multipath effects. Figure 2c is zoom-in view of Figure 2b.

The kinematic positions of the land-based vehicle are computed using the differential GNSS (DGNSS) technique. The GNSS instrumentation of the rover on top of the land-based vehicle was a NovAtel ANT-A72GOLA-TW, which can receive L1/L2 GPS and G1/G2 GLONASS signals. The pulse per second signal is also sent to the IMU for time synchronization.

**Figure 3:**
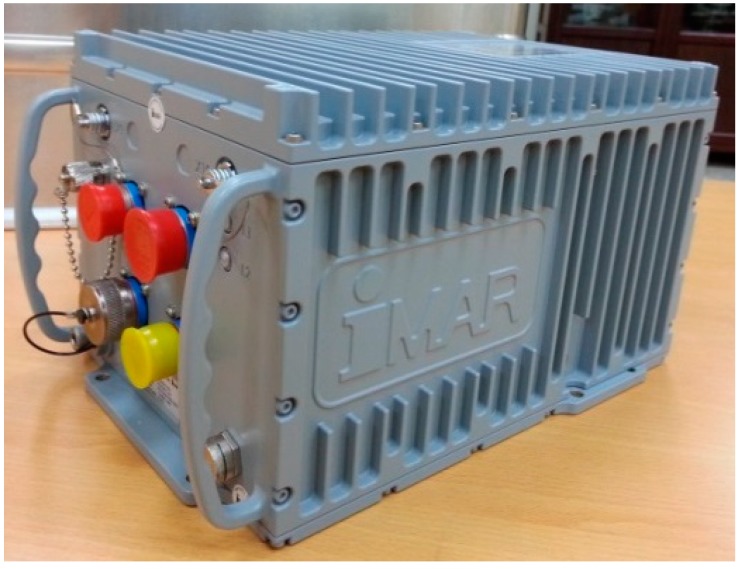
iNAV-RQH IMU.

The IMU is a strapdown navigation grade unit of type iNAV-RQH containing three QA2000 accelerometers and three GG1320 ring laser gyroscopes, as shown in Figure 3. The maximum output rate of iNAV-RQH is 300 Hz. Table 1 lists the main performance characteristics of both sensors.

**Table 1:** Performance characteristics of iNAV-RQH.

	QA2000 Accelerometer	GG1320 Ring Laser Gyroscope
Measurement Range	±2 g	±500 °/s
Non-linearity	$15\text{ μg}/{\text{g}}^{2}$	10 PPM
Scale Factor	70 PPM	10 PPM
Angular Random Walk	-	$0.0018\;{}^{\circ}/\sqrt{\text{h}}$
Acceleration Noise Density	$8\text{ μg}/\sqrt{\text{Hz}}$	-
Bias Repeatability	<15 μg	0.002 °/h

**Figure 4:**
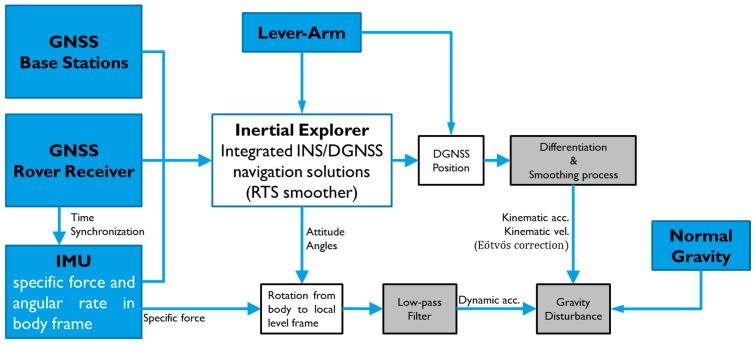
Data processing flowchart of the proposed SINS/GNSS gravimetry.

The flowchart for the estimation of gravity presented in this study is shown in Figure 4. The four main steps involved are described as follows.

First, the Inertial Explorer software from NovAtel was used to process the DGNSS mode and tightly-coupled integration using the RTS smoother. The optimal results of DGNSS positions and attitude angles were obtained for further processing.

Second, the kinematic accelerations were determined using the DGNSS positions based on a numerical differentiator. Numerical time-differentiation utilizes various discrete and analytic techniques. In this study, a fifth-order B-spline differentiator [19] was used to obtain kinematic accelerations. A smoothing process is necessary in the determination of kinematic accelerations because the numerical differentiation would increase the high frequency noises. Kincaid and Cheney [20] suggested the theory and model in numerical computations of the B-spline smoother and therefore, a third-order B-spline smoother with a window length of 180 s was adopted.

The raw data of accelerometers contain gravity and the effects of vehicle motion. IMU-measured dynamic accelerations were also processed by the same smoother, *i.e.*, third order B-spline smoother to remove high frequency errors of the data in the third step.

In the final step, gravity disturbance was computed by differencing the smoothed kinematic and dynamic accelerations. The biases of the accelerometers derived from the INS/GNSS RTS smoother were used in this step to remove the systematic errors directly. The standard deviations (STD) of the estimated biases in three components (X, Y and Z) are 9.60 mGal, 14.66 mGal and 7.12 mGal, respectively. In this way, reasonable and good estimations of gravity disturbance could be achieved.

## 5. Results and Discussion

Various scenarios and evaluations based on the purpose of this study were considered in the experiments and discussed in the following statements.

### 5.1. Performance of SINS/GNSS Gravimetry

**Figure 5:**
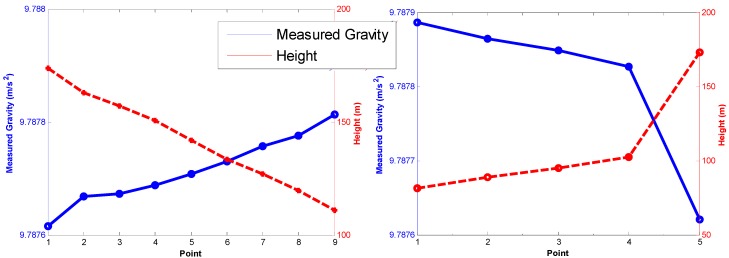
Relationship between the measured gravity and ellipsoidal height.

The performance of navigation grade iNAV-RQH was validated by first stopping the land-based vehicle at a point to perform Zero Velocity Update (ZUPT) and collect IMU and GNSS data in static mode for 3 to 5 min. A total of 209 ZUPT points were used in the experiment. During ZUPT periods, the motion of the vehicle was zero, and does not involve either kinematic acceleration or velocity. The IMU-measured dynamic accelerations represent the gravity vector. The errors of GNSS-derived kinematic accelerations and velocities would not affect the results of the calculated gravity. Figure 5 shows the two sections containing nine and five ZUPT points, respectively, selected for analysis because altitudes in these two sections have larger changes. Figure 5 illustrates that the correlation of measured gravity and elevation is highly negative, with a correlation coefficient of 0.957. Therefore, the measured gravity from iNAV-RQH shows reasonable results, with the mean gravity gradient about 0.283 mGal/m.

**Figure 6:**
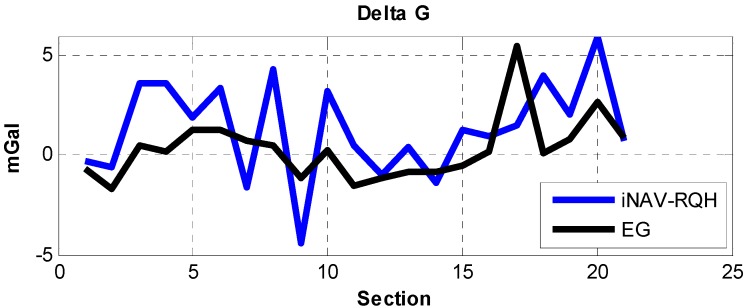
Comparison of the gravity at ZUPT points relative to the previous point derived from iNAV-RQH and Graviton EG.

An alternative method of assessing the accuracy of iNAV-RQH measured gravity is by employing the relative gravity next to the vehicle measured by using a Graviton EG relative gravimeter, an electronic version from LaCoste & Romberg. The repeatability of the Graviton EG is 0.001 mGal and 0.003 mGal in controlled and field conditions, respectively, and the absolute drift is smaller than 1 mGal per month. The contiguous 22 ZUPT points along the trajectory were selected and the gravity difference (Delta G) of the sections computed from Graviton EG and iNAV-RQH, as illustrated in Figure 6.

**Figure 7:**
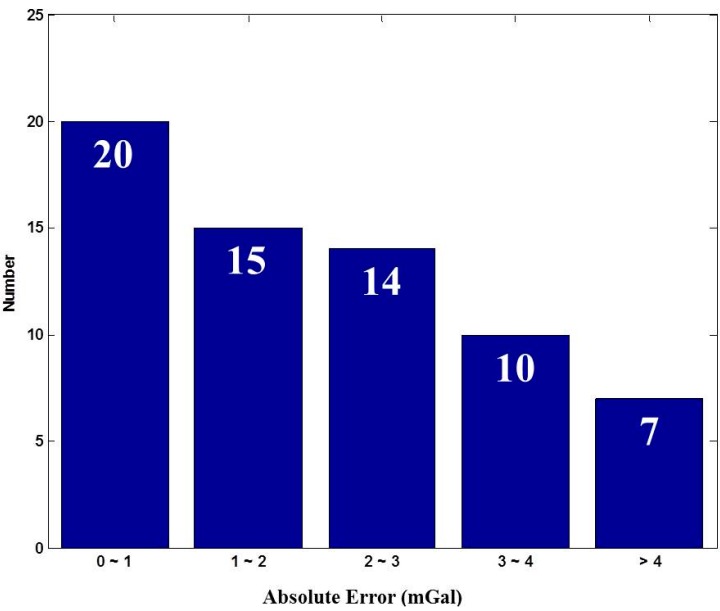
Statistical absolute error results.

The Delta G observed using the Graviton EG was assumed as the reference, and the absolute errors meaning the Delta G difference between the Delta G from iNAV-RQH and Graviton EG were calculated. Most of the absolute errors, as shown in Figure 7, range between 0 and 1 mGal. The mean of the absolute errors is 2.30 mGal with a standard deviation of 2.41 mGal. The comparison between the Delta G obtained through the gravimeter Graviton EG and through iNAV-RQH indicates that iNAV-RQH is capable of providing mGal-level gravity.

### 5.2. Determinations of Kinematic Velocity and Acceleration Using Multi-GNSS

As previously mentioned, the accuracy of the computed kinematic velocity and acceleration is crucial for estimating gravity using a moving base SINS/GNSS gravimetry. The multi-combination of GNSS data were collected at ZUPT points and processed using the DGNSS technique. The absence of errors in data and processing would be indicated by the computed kinematic velocity in horizontal and kinematic accelerations in the vertical being zero at ZUPT points.

**Figure 8:**
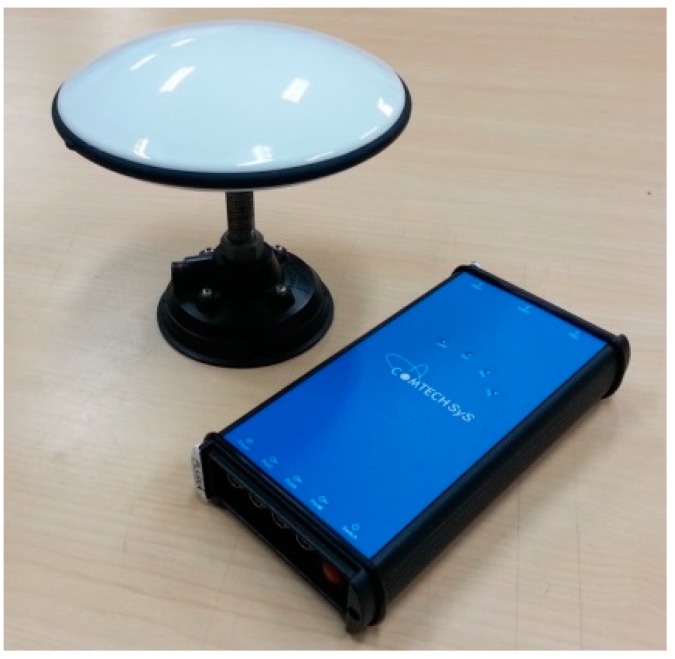
MB5000 GNSS antenna and receiver.

The testing GNSS receiver was an MB5000 GNSS Sensor from COMTECH, which was active with L1/L2 GPS, G1/G2 GLONASS, and B1/B2/B3 BDS signal. The exterior view is shown in Figure 8.

**Figure 9:**
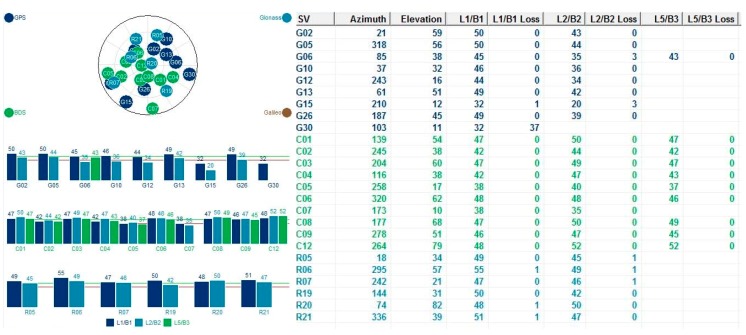
MB5000 software interface.

The DGNSS positioning results were processed using the Compass Solution software from COMTECH, and the static baseline mode was adopted. Two kinds of combined GNSS data were used in the study. The first dataset comprised a combination of GPS and GLONASS data for further computation, while the other dataset was a combination of GPS, GLONASS, and BDS. Based on the root mean square error (RMSE) of differences between the true value of zero and the computed kinematic acceleration, the kinematic acceleration derived from the data of three satellite systems indicated improvement of more than 11% compared with that from the two systems (Table 2). This improvement results from the higher availability of satellite measurements and better geometry from three satellite systems, which can elevate the accuracy of estimated gravity disturbance. The interface of the MB5000 software including the sky view and tracking satellites information is shown in Figure 9.

**Table 2:** Statistics of kinematic velocity and acceleration computed using different combinations of GNSS data.

	RMSE		
	$V_{e}$ ($10^{-5}\;m/s$)	$V_{n}$ ($10^{-5}\;m/s$)	$a_{u}$ (mGal)
**GPS + GLONASS**	14.05	13.01	3.91
**GPS + GLONASS + BDS**	11.62	7.28	3.47
	**Improvement (%)**		
**From two systems to three systems**	17.30	44.03	11.14

In this research, multi-combination of GNSS data observed by GPS, GLONASS, and BDS were used to evaluate the accuracies of the kinematic velocity and acceleration. The result shows obvious improvements, however, because the differential GNSS technique for kinematic data is not available so far, the multi-combination of GNSS data were not used to estimate gravity disturbances.

### 5.3. Feasibility Analysis for Groundwater Resource Detection

**Figure 10:**
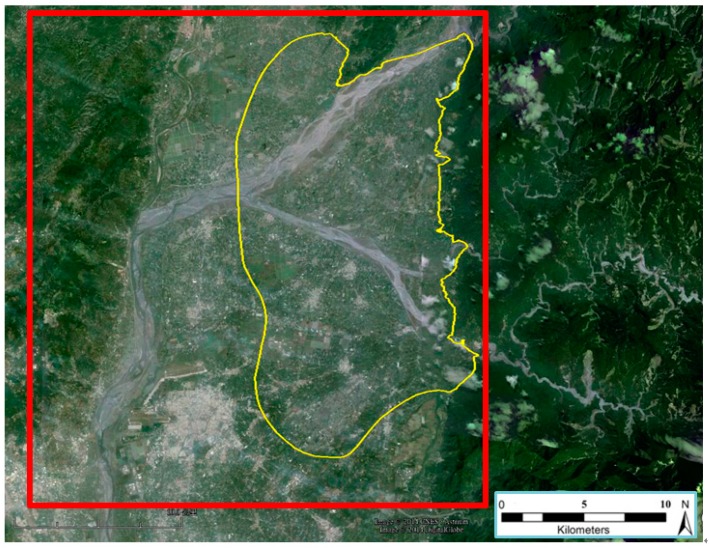
Experiment area at Pingtung Plain, Taiwan (background map courtesy of Google Earth).

The feasibility for groundwater resource detection using SINS/GNSS gravimetry was analyzed by selecting the Pingtung Plain in Taiwan (Figure 10) as the study area based on previous civil engineering and hydrology studies by the Taiwan Central Geological Survey [21]. The yellow line represents the potential area for water storage containing groundwater resources of approximately 1.2 ${\text{km}}^{3}$. The red zone is the experimental region, covering an area of about $30\times 30{\text{ km}}^{2}$.

**Figure 11:**
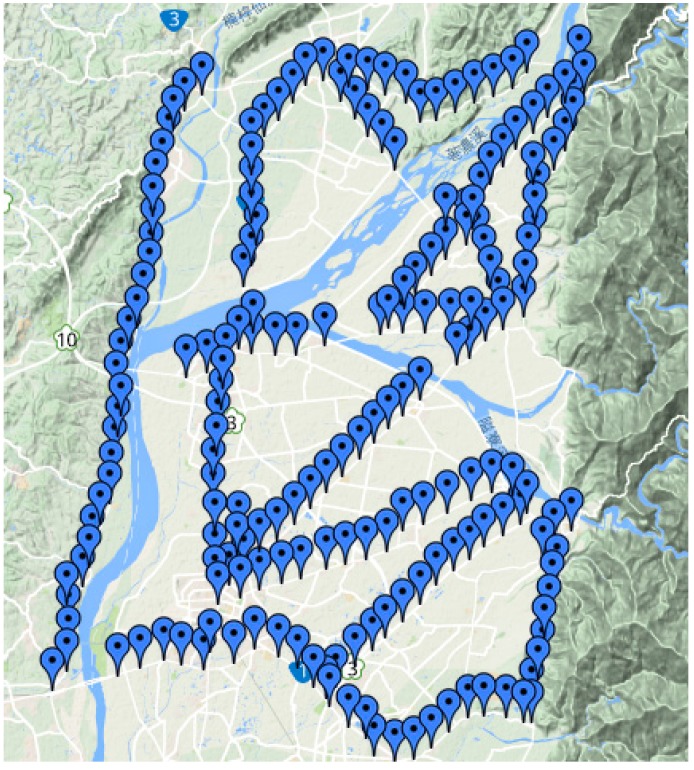
Distribution of ZUPT points (background map courtesy of Google Map).

The planned ZUPT were planted densely approximately every 1 km to ensure that better gravimetry results can be obtained and that the resolution of the test can be improved. The total number of ZUPT points was 209. Figure 11 shows the distribution of the ZUPT points.

Following Gerlach *et al.* [22], a fourth-order Butterworth low-pass filter with a cut-off frequency of 0.1 Hz was applied to process dynamic accelerations. In this study, both B-spline smoother and Butterworth filter were adopted to remove high frequency noises, and then the results obtained were compared. In Table 3, the averaged standard deviations (STD) show that the IMU-measured dynamic accelerations at ZUPT points processed by the B-spline smoother performed better because the high frequency noises were removed more accurately, allowing the gravity signals to stand out better. Therefore, the B-spline smoother is more effective and operative than the Butterworth filter and thus is used as the core filter of the developed SINS/GNSS gravimetry.

**Table 3:** Statistical results of different filters.

	Averaged STD of Dynamic Acc. (mGal)
**B-spline smoother**	4.84
**Butterworth filter**	107.70

The repeatability of the proposed land-based SINS/GNSS gravimetry can also be verified by collecting data twice from the same ZUPT point. The mean and standard deviation of the absolute difference in gravity disturbance can be calculated based on the entire repeated ZUPT points as listed in Table 4. As an indication of accuracy, the gravity disturbances at ZUPT points were examined after the smoothing process. The various extreme differences should be considered as outliers that do not fall within the three-sigma intervals. Finally, a total of 186 ZUPT points were obtained after excluding the large misfits. Table 4 also shows the significant improvement in the preliminary accuracy of the proposed land-based SINS/GNSS gravimetry.

**Table 4:** Accuracy of estimated gravity disturbances from the proposed system.

	Overall Result	Result After Excluding Large Misfits
**Mean (mGal)**	5.81	2.46
**STD (mGal)**	6.39	1.32

According to the size of potential area for Pingtung Plain [21], the equation used to compute gravity change caused by groundwater [23] can be expressed as follows:

(10) $$\text{g}=0.42\times {\text{P}}_{S}\times {\text{H}}_{\text{GW}}\times 10^{-3}$$

where g is gravity change (mGal) and ${\text{H}}_{\text{GW}}=175.1$ (m) is the groundwater level change of the potential area from empty to full, which is computed based on the groundwater recharge and area of Pingtung Plain. ${\text{P}}_{S}$ refers to soil porosity and the value ${\text{P}}_{S}=37$ that we adopted for further calculation was based on [24].

By considering the parameters of the Pingtung groundwater resource area, a variation in gravity of 2.72 mGal is determined according to Equation (9). The accuracy of the proposed land-based SINS/GNSS gravimetry after excluding the large outliers can be obtained and used for groundwater resource detection. The performances are based on an effective and appropriate smoothing process, which is crucial in reducing the high frequency noises.

## 6. Conclusions

In this research, a land-based SINS/GNSS gravimetry consisting of a navigation grade IMU iNAV-RQH and a GNSS receiver has been successfully developed. iNAV-RQH used as a gravimeter has an accuracy of 2.30 mGal compared with the results obtained from the Graviton EG relative gravimeter in stationary state. A comparison of the kinematic velocities and accelerations indicates that the use of a multi-combination of GNSS data yielded better results in the computation than the two-system option.

The proposed land-based system was also validated through several experiments, and a preliminary accuracy of the proposed land-based SINS/GNSS gravimetry with a standard deviation of 1.32 mGal was achieved after excluding any large outliers. A comparison with the gravity variation of 2.72 mGal resulting from the Pingtung groundwater in Taiwan shows that this system has potential for groundwater resource detection. The developed SINS/GNSS gravimetry is more feasible, effective and efficient to use for geodetic applications than using gravimeters and the spatial resolution of satellite missions.
